# Pharmacokinetics of BPA in Gliomas with Ultrasound Induced Blood-Brain Barrier Disruption as Measured by Microdialysis

**DOI:** 10.1371/journal.pone.0100104

**Published:** 2014-06-17

**Authors:** Feng-Yi Yang, Yi-Li Lin, Fong-In Chou, Yu-Chuan Lin, Yen-Wan Hsueh Liu, Lun-Wei Chang, Yu-Ling Hsieh

**Affiliations:** 1 Department of Biomedical Imaging and Radiological Sciences, National Yang-Ming University, Taipei, Taiwan; 2 Biophotonics and Molecular Imaging Research Center, National Yang-Ming University, Taipei, Taiwan; 3 Nuclear Science and Technology Development Center, National Tsing Hua University, Hsinchu City, Taiwan; 4 Institute of Nuclear Engineering and Science, National Tsing Hua University, Hsinchu City, Taiwan; Glasgow University, United Kingdom

## Abstract

The blood-brain barrier (BBB) can be transiently disrupted by focused ultrasound (FUS) in the presence of microbubbles for targeted drug delivery. Previous studies have illustrated the pharmacokinetics of drug delivery across the BBB after sonication using indirect visualization techniques. In this study, we investigated the in vivo extracellular kinetics of boronophenylalanine-fructose (BPA-f) in glioma-bearing rats with FUS-induced BBB disruption by microdialysis. After simultaneous intravenous administration of BPA and FUS exposure, the boron concentration in the treated brains was quantified by inductively coupled plasma mass spectroscopy. With FUS, the mean peak concentration of BPA-f in the glioma dialysate was 3.6 times greater than without FUS, and the area under the concentration-time curve was 2.1 times greater. This study demonstrates that intracerebral microdialysis can be used to assess local BBB transport profiles of drugs in a sonicated site. Applying microdialysis to the study of metabolism and pharmacokinetics is useful for obtaining selective information within a specific brain site after FUS-induced BBB disruption.

## Introduction

The blood-brain barrier (BBB) is a highly specialized endothelial structure occurring along the brain capillaries [1]. The transport of many drugs from the blood into the brain tissue is limited by the BBB. Several methods have been developed to circumvent this barrier in order to enhance drug delivery into the brain, such as the chemical modification of drugs, the osmotic opening of tight junctions, and the direct injection of the therapeutic agent or agents into the targeted brain area [2]. Recent studies have shown that focused ultrasound (FUS) can enhance the delivery of chemotherapeutic drugs into brain tumors and improve antitumor effects due to FUS-induced BBB disruption [3], [4]. As such, image-guided FUS technology may provide a novel strategy for targeted drug delivery in brain tumor treatment [5]. However, free drug concentrations cannot be measured if there is interference from signals produced by metabolites.

Patients with malignant gliomas have poor prognoses after radiation therapy and chemotherapy. Recently, several clinical studies have reported encouraging results in the treatment of patients with malignant brain tumors by boron neutron capture therapy (BNCT) [6]–[8]. For successful BNCT, sufficient neutrons and a selective accumulation of boron-10 (^10^B) must be delivered to the tumor site [9], [10]. The major challenge of boron delivery has been achieving sufficiently accurate tumor targeting to deliver therapeutic levels of boron to the tumor with minimal risk of toxicity to normal tissues. Therefore, chemical synthetic techniques and hyperosmotic BBB opening methods have been used to deliver the boron concentration into the brain tumor tissue for enhanced treatment efficacy [11]–[13]. However, these techniques produce dose-limiting side effects because they result in the boron concentration being increased throughout the whole brain.

The influx and efflux transport processes across the BBB are considered a key factor influencing the brain extracellular fluid (ECF) concentration of certain drugs. Intracerebral microdialysis is a tool to monitor the concentrations of free drugs and endogenous compounds at specific sites within the brain [14], [15]. Drug concentrations in brain dialysate reflect concentrations in brain ECF. Using intracerebral microdialysis, simultaneous measurements of the brain ECF concentration and the plasma concentration can produce useful information for evaluating the pharmacokinetics of drugs that have crossed the BBB. In a previous study, microdialysis was used to continuously assess the concentration of boronophenylalanine (BPA) in the ECF of the brain tumor tissue in patients undergoing BNCT for glioblastoma treatment [16]. The data showed that the uptake of BPA in the ECF of the tumor tissue was clearly higher than in the ECF of normal brains.

Previous studies have demonstrated that BPA-fructose (BPA-f) administration assisted by FUS can increase the accumulation of boron in brain tumors and elevate the tumor-to-normal-brain boron ratio [17], [18]. The purpose of this study was to evaluate the concentration-time profile of boron in brain tumors with FUS exposure after intravenous administration of BPA-f. Furthermore, the pharmacokinetics of boron in sonicated brain tumors were compared with those in non-sonicated brain tumors.

## Results

According to retrodialysis experiments, the in vivo relative recoveries were determined to be 48±7% (n = 3) for the plasma vascular probe and 30±5% (n = 4) for the brain tumor probe. The concentrations of BPA measured in the physiological samples were corrected for the relative recovery of the probe used.

**Figure 1:**
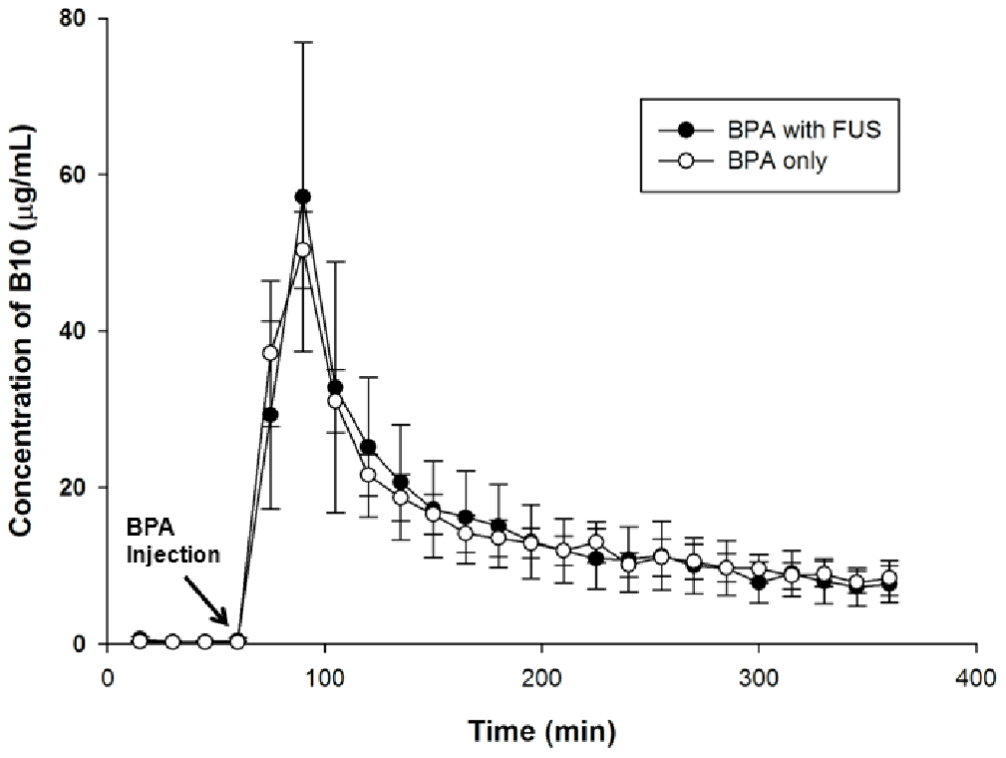
The boron concentration versus time profiles in the dialysate of the plasma after BPA administration at 500/kg in glioma-bearing rats with or without FUS exposure. The concentration of boron measured in each 15-min sample of plasma represents the average concentration of that interval. Data are expressed as mean ± SEM, n = 6.

The concentration versus time curves for boron in plasma before and after intravenous injection both with and without FUS exposure were almost identical (Fig. 1). The mean peak values were similar to those of the concentration profiles, in which the highest boron accumulation in the brain tumor ECF occurred at 30 min after drug injection and declined rapidly during the elimination phase.

**Figure 2:**
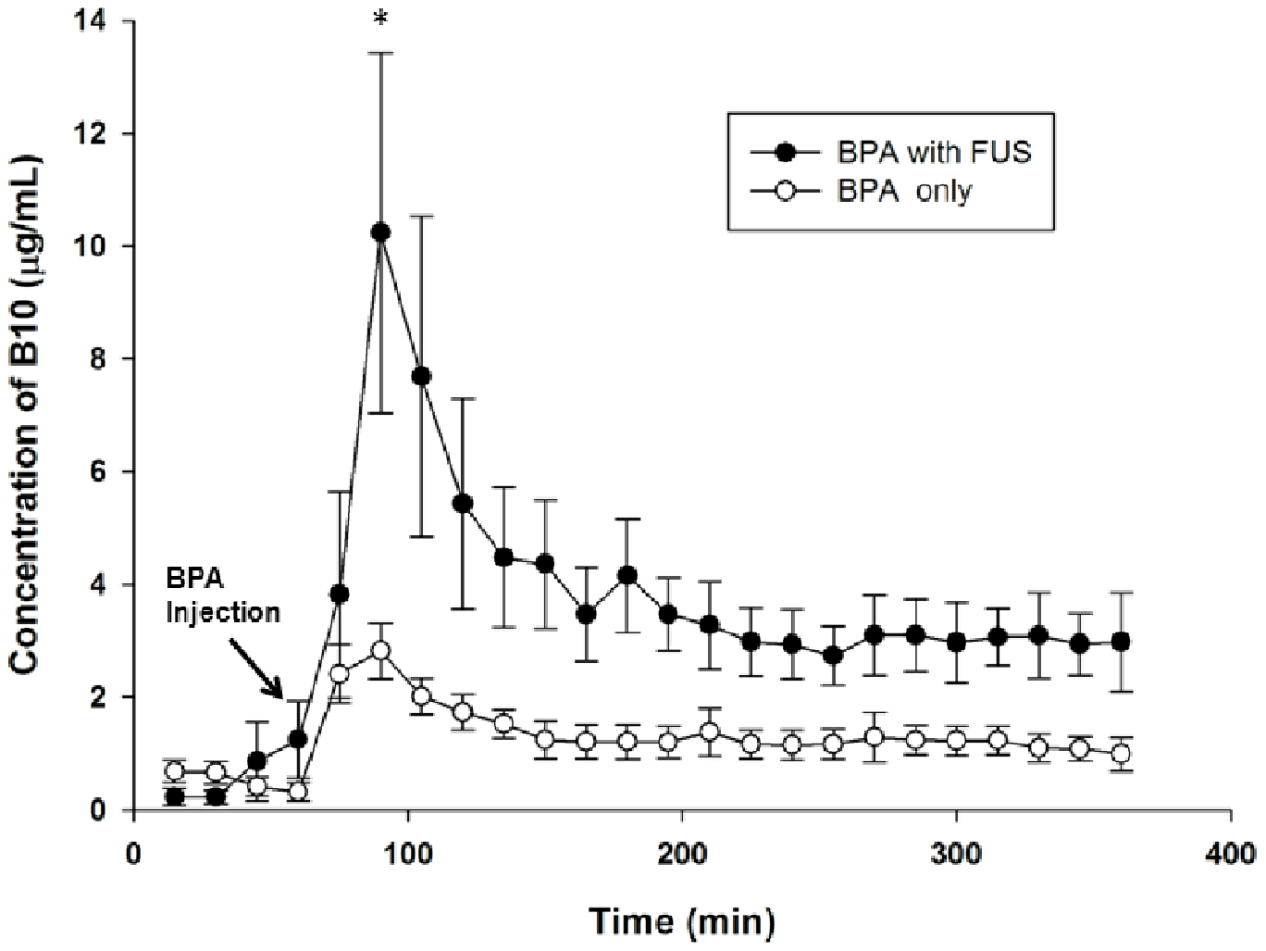
Representative data for boron concentration versus time profiles in tumor ECF after BPA administration at 500/kg in glioma-bearing rats with or without FUS exposure. The concentration of boron measured in each 15-min sample of tumor ECF represents the average concentration of that interval. Data are expressed as mean ± SEM, n = 5. (*p<0.05).

The concentration versus time curves for boron in the brain tumor with and without FUS exposure after BPA (500 mg/kg) administration are shown in Fig. 2. The concentrations of boron in brain tumor dialysates were determined from the calibration curve. No significant difference in boron concentrations in the sonicated tumors and control tumors was found before intravenous bolus injection of BPA-f. Compared to the control tumors, a significant accumulation of boron can be seen in the sonicated tumors for a period of 5 h after BPA-f injection. The concentrations of boron in both types of tumors reached a peak at 30 min after drug administration and then declined rapidly. Furthermore, the mean peak value of boron in the ECF of the sonicated tumors was ∼3.63-fold higher than in control tumor ECF.

Tables 1 and 2 show the pharmacokinetic parameters of boron with or without sonication in plasma and tumor ECF, respectively. Table 1 shows that no significant differences were found for the four parameters in plasma after FUS-induced BBB disruption as compared to the parameters for the BPA-alone group. Table 2 indicates that the maximum tumor ECF concentration (*Ct*_max_), MRT, and area under the concentration versus time curve (AUC) values clearly increased after FUS exposure as compared with those values for the BPA administration only group. The increases in MRT (from 313 to 503) and AUC/dose (from 1.8 to 3.8) show that the retention time of BPA in the tumor was extended with FUS exposure.

**Table 1:** Pharmacokinetic Parameters of BPA in Plasma after Intravenous Injection.

Parameters	BPA 500 mg/kg only	BPA 500 mg/kg with FUS
*Cp*_max_ (µg/mL)	53.0±6.6	70.8±12.0
MRT (min)	208±29	209±46
AUC (min µg/mL)	6412±1030	7210±1430
AUC/dose	12.8±2.1	14.4±2.9

*Cp*_max_: maximum plasma concentration (*n* = 6 for each group).

MRT: mean residence time.

AUC: area under the concentration.

**Table 2:** Pharmacokinetic Parameters of BPA in Tumor ECF after Intravenous Injection.

Parameters	BPA 500 mg/kg only	BPA 500 mg/kg with FUS
*Ct*_max_ (µg/mL)	3.8±0.5	6.8±1.9
MRT (min)	313±87	503±192
AUC (min µg/mL)	888±225	1887±600
AUC/dose	1.8±0.5	3.8±1.2

*Ct*_max_: the maximum tumor ECF concentration (*n* = 5 for each group).

MRT: mean residence time.

AUC: area under the concentration.

## Discussion

BPA has been applied as a potential boron carrier in clinical trials with BNCT. BPA conjugated with fructose has been proven to enhance drug accumulation in the tumor due to increased solubility [19]. Non-invasive methods used for the in vivo determination of the local pharmacokinetics of a drug within the brain have been revolutionized by the development of positron emission tomography (PET) and nuclear magnetic resonance spectroscopy. In a previous study, PET was proposed to assess the biodistribution of ^18^F-labeled BPA-f [20].^ 18^F-FBPA-f showed specific tumor uptake in F98 glioma-bearing rats, indicating that PET can be used as a probe for BPA-f in BNCT [21]. Nevertheless, the key limitation of PET scanning is the instability of the isotope, and no free drug concentrations can be obtained. Furthermore, the spatial resolution of non-invasive PET imaging techniques is limited.

Since gliomas are highly infiltrative tumors, it actually would be highly desirable to have boron delivery to infiltrating tumor cells [22]. The barrier function of the BBB can become altered after FUS exposure. Thus, the further study as to whether FUS can enhance delivery to these infiltrating tumor cells in the F98 glioma model are needed to be investigated. Many studies have investigated the pharmacokinetics of BBB permeability in brains after FUS-induced BBB disruption using dynamic contrast-enhanced (DCE)-MRI [23]–[25]. In addition, ^99m^Tc-DTPA micro-SPECT/CT shows that FUS not only increases the permeability of the BBB significantly at the sonicated site, but also elevates the lesion-to-normal brain ratio significantly in the focal region [26]. However, non-invasive imaging can provide neither chemical information nor information on the metabolites. Although intracerebral microdialysis has the disadvantage of being invasive, microdialysis sampling coupled to an appropriate analytical model can be used to study pharmacokinetics and metabolism in the target site. This technique has a temporal and spatial resolution suitable for evaluating neuropharmacokinetics in the brain ECF following drug administration with FUS exposure. Thus, such data may facilitate knowledge of the temporal features of pharmacological change after FUS-induced BBB disruption.

To our knowledge, this is the first *in vivo* study evaluating the local BBB transport profile in tumor ECF during FUS exposure. The BBB functionality is not significantly influenced by microdialysis probe implantation when experimental procedures are well-controlled. The results (Fig. 2) showed that FUS exposure increased the peak tumor ECF uptake value (10.23±3.19 µg/mL) while resulting in the same peak uptake time (at 30 min after BPA injection) compared with the control tumor (2.82±0.49 µg/mL). After FUS-induced BBB disruption, the boron concentration in tumor ECF followed the concentration in blood (Fig. 1), suggesting that neutron irradiation should be delivered as closely as possible to the time when the peak blood level is reached. In terms of the pharmacokinetic parameters (Table 2), the increases of *Ct*_max_ (from 3.8 to 6.8), MRT (from 313 to 503), and AUC/dose (from 1.8 to 3.8) imply that the absorption of BPA in the brain tumor was enhanced by FUS exposure. The major strength of intracerebral microdialysis lies in the fact that it not only measures the drug concentrations, but also monitors metabolite concentrations selectively within the targeted brain site. Thus, further investigations exploring the metabolism of BPA-f in the brain should be feasible.

This study demonstrated that the microdialysis technique is useful for the assessment of pharmacokinetics during FUS-induced BBB disruption while also not causing any severe side effects. The uptake of boron in the sonicated tumor ECF was clearly higher than that in the control tumor ECF, reaching a maximum of 3.63 times higher. The peak boron concentration in sonicated tumor ECF was reached at 30 min after BPA-f injection and at the same time as in blood. The boron concentration versus time profile in blood could thus be a good indicator of the optimal time for neutron irradiation when FUS exposure is applied in a brain tumor during BNCT.

## Materials and Methods

### Glioma Brain Tumors

Male Fischer 344 rats (11–13 wk, 250–280 g) were anesthetized with an intraperitoneal injection of pentobarbital at a dose of 40 mg/kg of body weight. Then, 1×10^5^ F98 rat glioma cells (a generous gift from Dr. Rolf F. Barth, Ohio State University) in 10 µL Hanks’ balanced salt solution without Mg^2+^ and Ca^2+^ were stereotactically injected into the right hemisphere (5.0 mm posterior and 3.0 mm lateral to the bregma) of each rat’s brain at a depth of 5.0 mm from the brain surface. Finally, the hole in the skull was sealed with bone wax, and the wound was flushed with iodinated alcohol. The F98 rat glioma has been described in previous study [27]. All *in vivo* experiments were performed according to ARRIVE guidelines and were approved by the Animal Care and Use Committee of the National Yang-Ming University.

### Focused Ultrasound Exposure

FUS was produced by a 1 MHz single-element focused transducer (A392S, Panametrics, Waltham, MA, USA) with a diameter of 38 mm and a radius of curvature of 63.5 mm. The half-maximum of the pressure amplitude of the focal zone had a diameter and length of 3 mm and 26 mm, respectively. The transducer was mounted on a removable cone filled with degassed water, and its tip was sealed with a polyurethane membrane. The FUS system setup was the same as described previously [28]. Animals were anesthetized intraperitoneally with urethane (1.2 g/kg) prior to the experiments. Ultrasound contrast agent (UCA, SonoVue, Bracco International, Amsterdam, the Netherlands) was injected into the femoral vein of the rats approximately 15 s before each sonication. The transducer was applied with a burst length of 50 ms at a 5% duty cycle and a repetition frequency of 1 Hz. The sonication time was 60 s at an acoustic power of 2.86 W and a UCA dose of 300 µL/kg. Ultrasound exposure was delivered to the tumor site on day 10 after tumor cell implantation.

### Microdialysis

**Figure 3:**
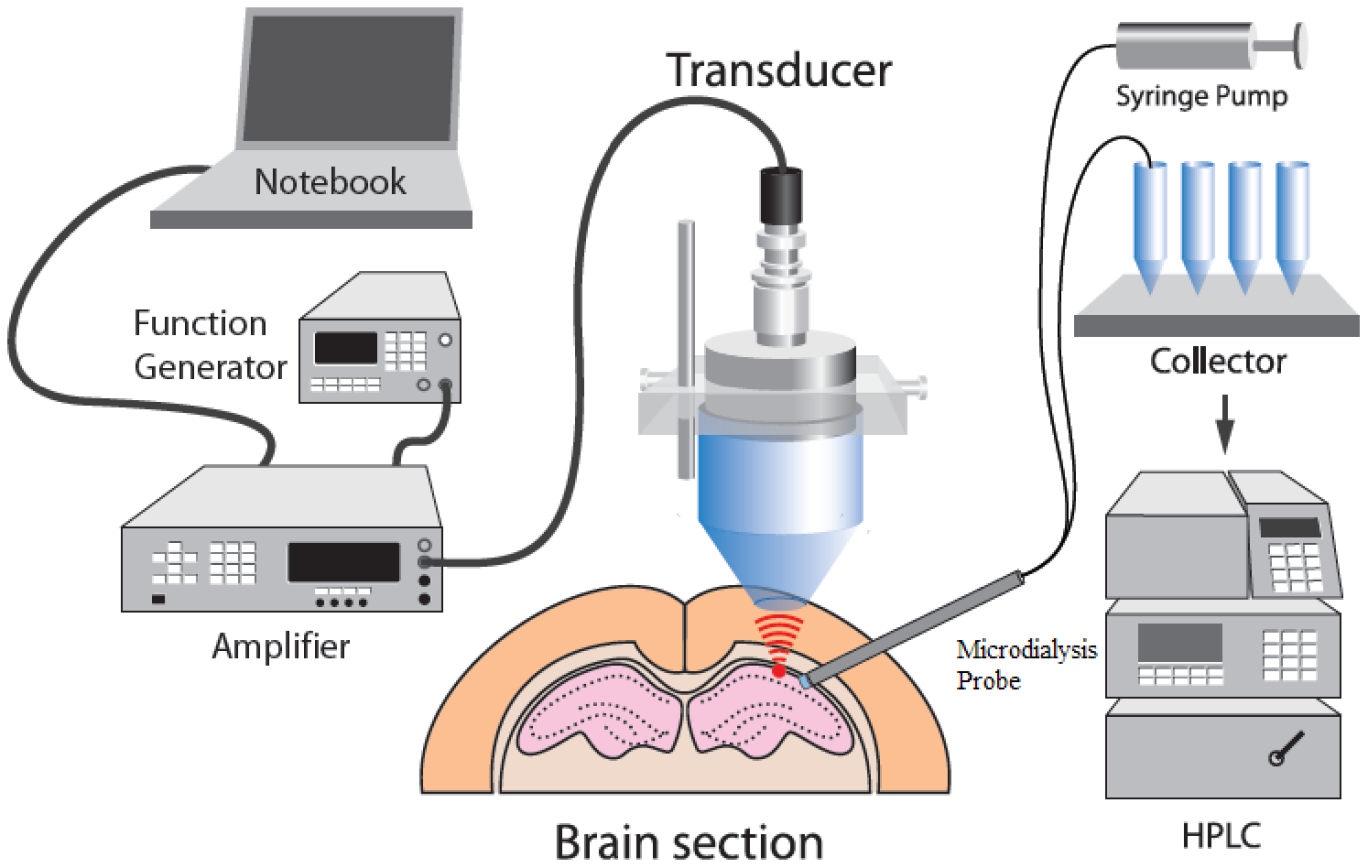
Schematic diagram illustrating the experimental setup for FUS and microdialysis.

To evaluate the variability of BPA concentration within the brain tumor, a commercially available microdialysis probe (MAB 6, Stockholm, Sweden) was applied to sample the unbound BPA in the rat brain. Simultaneously, another microdialysis probe (MAB 7, Stockholm, Sweden) was implanted into the jugular vein toward the rat’s right atrium. For brain ECF sampling, the rat was mounted on a stereotaxic frame. An incision was made in the scalp, and a small hole was drilled on the right side of the skull for implantation of the rigid microdialysis probe in the tumor. After implantation of the microdialysis probes, the probes were perfused with Ringer’s solution (147 mM sodium chloride, 4 mM potassium chloride, 2.2 mM calcium chloride) by a microinjection pump (CMA 402, Stockholm, Sweden) at a flow rate of 2 µL/min. Dialysate samples were collected using a microfraction collector (MAB 85, Stockholm, Sweden). The entire experimental system is shown in Fig. 3.

**Figure 4:**
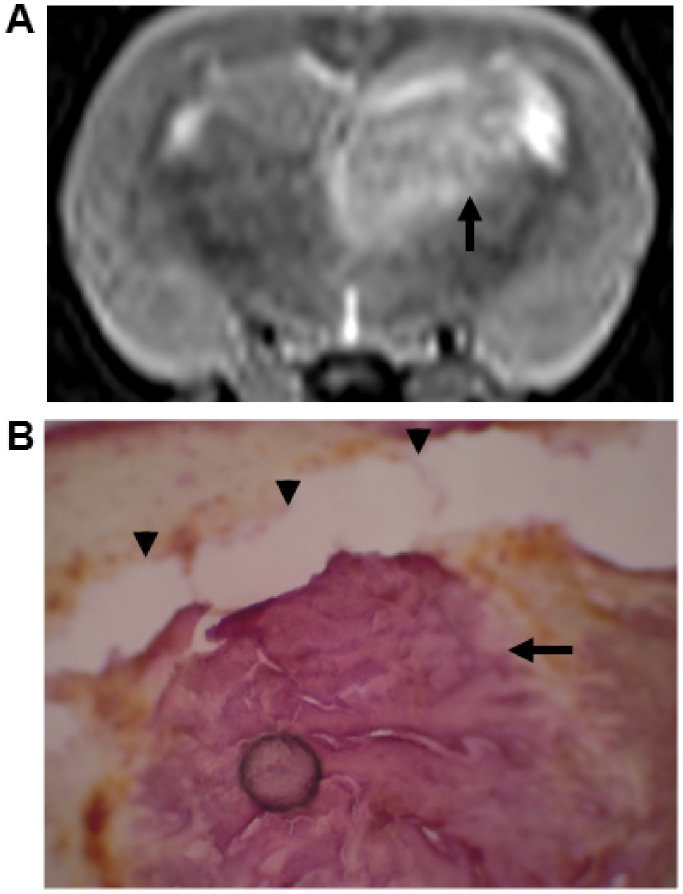
Tumor growth was monitored by MRI and histology. (A) Representative sample of tumor (arrow) by T2-weighted MR images on day 10 after implantation. (B) Hematoxylin-eosin-stained section shows the tunnel (arrow heads) punctured by the probe within the tumor tissue (arrow).

Tumor progression was monitored by means of T2-weighted MR images (TRIO 3-T MRI, Siemens MAGNETOM, Germany) obtained on day 10 after tumor implantation (Fig. 4A). The maximum tumor size was about 0.125 cm^3^. Moreover, the track of the probe within the tumor was verified by hematoxylin and eosin (H&E) staining (Thermo-Scientific, Waltham, MA, USA) (Fig. 4B) following microdialysis experiment. At the time of sacrifice, all rats were deeply anesthetized under isoflurane anesthesia (5%). After probe placement, 2 h were allowed to elapse so that probe and tumor ECF could equilibrate [29], [30]. Subsequently, 24 sets of microdialysis samplings of tumor ECF and plasma were performed every 15 min for 6 h before and after intravenous bolus injection of BPA-fr (Taiwan Biotech Co., LTD., Taoyuan, Taiwan) at a dose of 500 mg/kg. These 24 sets of microdialysis samplings consisted of the four sets of samplings taken prior to BPA-fr injection. Boron concentration in the tumor ECF and plasma were assayed by inductively coupled plasma mass spectroscopy (ICP-MS) and calibrated with the standard curve derived from the measurement of boric acid standard.

### Recovery of the Microdialysate

To determine the unbound BPA concentration in the tumor ECF and plasma from the microdialysis data, the concentration of BPA in the microdialysis samples must be adjusted. A retrodialysis technique was used for the experiment of in vivo recovery [31]. One hour after probe implantation, Ringer’s solution containing BPA (350 µg/mL) was perfused through the probes and separately into the rat’s blood and brain at a constant rate of 2 µL/min. Furthermore, the dialysate (*C*_dial_) and perfusate (*C*_perf_) concentrations of BPA were measured by ICP-MS. The relative in vivo recovery of BPA across the dialysis membrane was calculated using the following equation: *R*_dial = _1-*C*_dial_/*C*_perf_, where *R*_dial_ is the in vivo BPA recovery.

### Pharmacokinetics

The pharmacokinetic parameters of boron in the plasma and tumor ECF were calculated for each set of sampled data using noncompartmental methods in the WinNonlin Version 5.2.1 software program (Pharsight, Montreal, Quebec, Canada). The area under the concentration versus the time curve extrapolated to infinity (AUC_0-∞_) was calculated using log-trapezoidal methods. Maximum observed concentration (*C*_max_) and time to maximum concentration values were obtained directly from concentration versus time profiles. Mean residence time (MRT) extrapolated to infinity was calculated as AUMC_0-∞_/AUC_0-∞_−(infusion time)/2, where AUMC_0-∞_ is the area under the first moment curve extrapolated to infinity.

### Histological Observation

Two glioma-bearing rats were prepared for histological examination. The rats were perfused with saline and 10% neutral buffered formalin. The brains were removed, embedded in paraffin, and then serially sectioned into 30-µm-thick slices. The slices were stained with H&E to visualize their general cellular structure. Photomicrographs of 5 µm-thicknesses of the H&E stained tissues were obtained using a Mirax Scan digital microscope slide scanner (Carl Zeiss, Mirax 3D Histech) with a Plan-Apochromatic 20/0.8 objective lens.

### Statistical Analysis

All data are shown as mean ± SEM. Data analysis was performed using an unpaired Student *t* test. Statistical significance was noted if *p* ≤ 0.05.

## References

[1] Rubin LL, Staddon JM (1999) The cell biology of the blood-brain barrier. Annu Rev Neurosci 22: 11–28. 10202530 10.1146/annurev.neuro.22.1.11

[2] Kroll RA, Neuwelt EA (1998) Outwitting the blood-brain barrier for therapeutic purposes: osmotic opening and other means. Neurosurgery 42: 1083–1099 discussion 1099–1100. 9588554 10.1097/00006123-199805000-00082

[3] Yang FY, Teng MC, Lu M, Liang HF, Lee YR, et al (2012) Treating glioblastoma multiforme with selective high-dose liposomal doxorubicin chemotherapy induced by repeated focused ultrasound. Int J Nanomedicine 7: 965–974. 22393293 10.2147/IJN.S29229 PMC3289450

[4] Yang FY, Wong TT, Teng MC, Liu RS, Lu M, et al (2012) Focused ultrasound and interleukin-4 receptor-targeted liposomal doxorubicin for enhanced targeted drug delivery and antitumor effect in glioblastoma multiforme. J Control Release 160: 652–658. 22405901 10.1016/j.jconrel.2012.02.023

[5] Hynynen K, McDannold N, Vykhodtseva N, Jolesz FA (2001) Noninvasive MR imaging-guided focal opening of the blood-brain barrier in rabbits. Radiology 220: 640–646. 11526261 10.1148/radiol.2202001804

[6] Skold K, Gorlia T, Pellettieri L, Giusti V, B HS, et al (2010) Boron neutron capture therapy for newly diagnosed glioblastoma multiforme: an assessment of clinical potential. Br J Radiol 83: 596–603. 20603410 10.1259/bjr/56953620 PMC3473677

[7] Miyatake S, Kawabata S, Yokoyama K, Kuroiwa T, Michiue H, et al (2009) Survival benefit of Boron neutron capture therapy for recurrent malignant gliomas. J Neurooncol 91: 199–206. 18813875 10.1007/s11060-008-9699-x

[8] Kawabata S, Miyatake S, Kuroiwa T, Yokoyama K, Doi A, et al (2009) Boron neutron capture therapy for newly diagnosed glioblastoma. J Radiat Res 50: 51–60. 18957828 10.1269/jrr.08043

[9] Barth RF, Soloway AH, Brugger RM (1996) Boron neutron capture therapy of brain tumors: past history, current status, and future potential. Cancer Invest 14: 534–550. 8951358 10.3109/07357909609076899

[10] Barth RF, Coderre JA, Vicente MG, Blue TE (2005) Boron neutron capture therapy of cancer: current status and future prospects. Clin Cancer Res 11: 3987–4002. 15930333 10.1158/1078-0432.CCR-05-0035

[11] Barth R, Yang W, Rotaru J, Moeschberger M, Joel D, et al (1997) Boron neutron capture therapy of brain tumors: enhanced survival following intracarotid injection of either sodium borocaptate or boronophenylalanine with or without blood-brain barrier disruption. Cancer Research 57: 1129. 9067283

[12] Barth R, Yang W, Rotaru J, Moeschberger M, Boesel C, et al (2000) Boron neutron capture therapy of brain tumors: enhanced survival and cure following blood-brain barrier disruption and intracarotid injection of sodium borocaptate and boronophenylalanine. International Journal of Radiation Oncology* Biology* Physics 47: 209–218. 10.1016/s0360-3016(00)00421-1 10758326

[13] Barth RF, Yang W, Bartus RT, Rotaru JH, Ferketich AK, et al (2002) Neutron capture therapy of intracerebral melanoma: enhanced survival and cure after blood-brain barrier opening to improve delivery of boronophenylalanine. Int J Radiat Oncol Biol Phys 52: 858–868. 11849812 10.1016/s0360-3016(01)02734-1

[14] Westerink BH, Damsma G, Rollema H, De Vries JB, Horn AS (1987) Scope and limitations of in vivo brain dialysis: a comparison of its application to various neurotransmitter systems. Life Sci 41: 1763–1776. 2889121 10.1016/0024-3205(87)90695-3

[15] Benveniste H, Huttemeier PC (1990) Microdialysis–theory and application. Prog Neurobiol 35: 195–215. 2236577 10.1016/0301-0082(90)90027-e

[16] Bergenheim AT, Capala J, Roslin M, Henriksson R (2005) Distribution of BPA and metabolic assessment in glioblastoma patients during BNCT treatment: a microdialysis study. J Neurooncol 71: 287–293. 15735919 10.1007/s11060-004-1724-0

[17] Yang FY, Chen YW, Chou FI, Yen SH, Lin YL, et al (2012) Boron neutron capture therapy for glioblastoma multiforme: enhanced drug delivery and antitumor effect following blood-brain barrier disruption induced by focused ultrasound. Future Oncol 8: 1361–1369. 23130933 10.2217/fon.12.118

[18] Alkins RD, Brodersen PM, Sodhi RN, Hynynen K (2013) Enhancing drug delivery for boron neutron capture therapy of brain tumors with focused ultrasound. Neuro Oncol 15: 1225–1235. 23640533 10.1093/neuonc/not052 PMC3748911

[19] Yoshino K, Suzuki A, Mori Y, Kakihana H, Honda C, et al (1989) Improvement of solubility of p-boronophenylalanine by complex formation with monosaccharides. Strahlenther Onkol 165: 127–129. 2928932

[20] Kabalka GW, Nichols TL, Smith GT, Miller LF, Khan MK, et al (2003) The use of positron emission tomography to develop boron neutron capture therapy treatment plans for metastatic malignant melanoma. J Neurooncol 62: 187–195. 12749713 10.1007/BF02699944

[21] Wang H, Liao A, Deng W, Chang P, Chen J, et al (2004) Evaluation of 4-borono-2–18F-fluoro-L-phenylalanine-fructose as a probe for boron neutron capture therapy in a glioma-bearing rat model. Journal of Nuclear Medicine 45: 302. 14960653

[22] Smith DR, Chandra S, Barth RF, Yang W, Joel DD, et al (2001) Quantitative imaging and microlocalization of boron-10 in brain tumors and infiltrating tumor cells by SIMS ion microscopy: relevance to neutron capture therapy. Cancer Res 61: 8179–8187. 11719448

[23] Vlachos F, Tung YS, Konofagou EE (2010) Permeability assessment of the focused ultrasound-induced blood-brain barrier opening using dynamic contrast-enhanced MRI. Phys Med Biol 55: 5451–5466. 20736501 10.1088/0031-9155/55/18/012 PMC4005850

[24] Vlachos F, Tung YS, Konofagou E (2011) Permeability dependence study of the focused ultrasound-induced blood-brain barrier opening at distinct pressures and microbubble diameters using DCE-MRI. Magn Reson Med 66: 821–830. 21465543 10.1002/mrm.22848 PMC3919956

[25] Park J, Zhang Y, Vykhodtseva N, Jolesz FA, McDannold NJ (2012) The kinetics of blood brain barrier permeability and targeted doxorubicin delivery into brain induced by focused ultrasound. J Control Release 162: 134–142. 22709590 10.1016/j.jconrel.2012.06.012 PMC3520430

[26] Yang FY, Wang HE, Lin GL, Teng MC, Lin HH, et al (2011) Micro-SPECT/CT-based pharmacokinetic analysis of 99mTc-diethylenetriaminepentaacetic acid in rats with blood-brain barrier disruption induced by focused ultrasound. J Nucl Med 52: 478–484. 21321259 10.2967/jnumed.110.083071

[27] Barth RF (1998) Rat brain tumor models in experimental neuro-oncology: the 9L, C6, T9, F98, RG2 (D74), RT-2 and CNS-1 gliomas. J Neurooncol 36: 91–102. 9525831 10.1023/a:1005805203044

[28] Yang FY, Lin YS, Kang KH, Chao TK (2011) Reversible blood-brain barrier disruption by repeated transcranial focused ultrasound allows enhanced extravasation. J Control Release 150: 111–116. 21070825 10.1016/j.jconrel.2010.10.038

[29] Johansen MJ, Newman RA, Madden T (1997) The use of microdialysis in pharmacokinetics and pharmacodynamics. Pharmacotherapy 17: 464–481. 9165551

[30] Kehr J (1993) A survey on quantitative microdialysis: theoretical models and practical implications. J Neurosci Methods 48: 251–261. 8105153 10.1016/0165-0270(93)90096-a

[31] Evrard PA, Deridder G, Verbeeck RK (1996) Intravenous microdialysis in the mouse and the rat: development and pharmacokinetic application of a new probe. Pharm Res 13: 12–17. 8668659 10.1023/a:1016056628685

